# Converging on Bladder Health through Design Thinking: From an Ecology of Influence to a Focused Set of Research Questions

**DOI:** 10.3390/ijerph17124340

**Published:** 2020-06-17

**Authors:** Jessica B. Lewis, Sonya S. Brady, Siobhan Sutcliffe, Ariana L. Smith, Elizabeth R. Mueller, Kyle Rudser, Alayne D. Markland, Ann Stapleton, Sheila Gahagan, Shayna D. Cunningham, Prevention of Lower Urinary Tract Symptoms (PLUS) Research Consortium

**Affiliations:** 1 Department of Chronic Disease Epidemiology, Yale School of Public Health, New Haven, CT 06520, USA; 2 Division of Epidemiology and Community Health, University of Minnesota School of Public Health, Minneapolis, MN 55454, USA; ssbrady@umn.edu; 3 Department of Surgery, Division of Public Health Sciences, Washington University School of Medicine, St. Louis, MO 63110, USA; sutcliffes@wustl.edu; 4 Department of Surgery, Division of Urology, Perelman School of Medicine, University of Pennsylvania, Philadelphia, PA 19104, USA; Ariana.Smith@pennmedicine.upenn.edu; 5 Division of Female Pelvic Medicine and Reconstructive Surgery, Stritch School of Medicine, Loyola University Chicago, Loyola University Medical Center, Maywood, IL 60153, USA; emuelle@lumc.edu; 6 Division of Biostatistics, University of Minnesota School of Public Health, Minneapolis, MN 55455, USA; rudser@umn.edu; 7 Department of Medicine, Division of Gerontology, Geriatrics, and Palliative Care, School of Medicine, University of Alabama and the Geriatric Research, Education, and Clinical CenterBirmingham, AL 35233, USA; amarkland@uabmc.edu; 8 Department of Medicine, Division of Allergy and Infectious Disease, University of Washington, Seattle, WA 98195, USA; stapl@uw.edu; 9 Department of Pediatrics, University of California, San Diego, CA 92161, USA; sgahagan@pedsmail.ucsd.edu; 10 Department of Social and Behavioral Sciences, Yale School of Public Health, New Haven, CT 06520, USA; shayna.cunningham@yale.edu; 11 Division of Biostatistics, School of Public Health, University of Minnesota, Minneapolis, MN 55455, USA; plus-ops@umn.edu

**Keywords:** lower urinary tract symptom, design thinking, prevention, bladder health, transdisciplinary, public health, women, research question development

## Abstract

Lower urinary tract symptoms affect a substantial number of women in the United States (U.S.) and globally. In 2015, the Prevention of Lower Urinary tract Symptoms in women (PLUS) Research Consortium was funded to establish the scientific basis for prevention efforts by (1) understanding healthy bladder function and (2) identifying risk and protective factors for bladder health in women across the lifecourse. This transdisciplinary consortium generated a list of over 600 candidate risk and protective factors for bladder health in women and girls and refined and prioritized these into 29 focused research questions to inform a national longitudinal observational study in the U.S. This paper describes that process using design thinking, a human-centered set of principles and strategies by which innovations are developed, as a framework. Design thinking is an iterative process consisting of five stages: Empathizing with end-users of innovations, Defining core principles girding the work, Ideation of all possible solutions, and rapid-cycle Prototyping and Testing of solutions. Lessons learned are offered to inform future prevention science research endeavors that might benefit from such an approach.

## 1. Introduction

Lower urinary tract symptoms (LUTS) affect a substantial number of women and girls in the United States (U.S.) and across the globe [1,2,3,4,5,6,7,8]. Nearly one in four women over the age of 30 in the U.S. experience poor bladder health, based on the frequency and impact of LUTS, with increasing prevalence over the lifecourse [6]. Much research on LUTS in women and girls has focused on identifying and treating those who are already suffering from symptoms. In 2015, the U.S. National Institute of Diabetes and Digestive and Kidney Diseases (NIDDK) funded a transdisciplinary scientific initiative to create a prevention research agenda for LUTS in women and girls [9]. The Prevention of Lower Urinary tract Symptoms (PLUS) Research Consortium brings together investigators from seven universities and a scientific and data coordinating center (SDCC) with expertise in over 60 disciplinary areas, including medicine, nursing, public health, psychology, sociology, epidemiology, social welfare, anthropology, biostatistics, community engagement, and prevention science [10]. The charge of the PLUS Consortium was to establish the scientific basis for future prevention intervention studies by (1) developing a better understanding of healthy bladder function and (2) identifying risk and protective factors for bladder health in U.S. women across the lifecourse [9].

**Figure 1:**
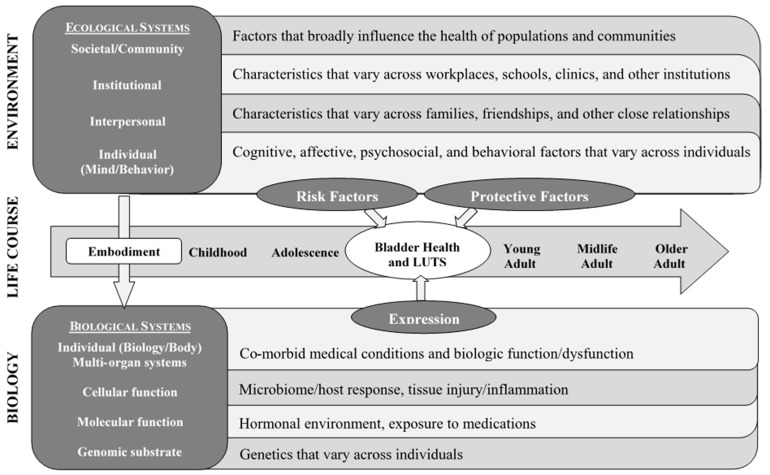
Prevention of Lower Urinary tract Symptoms (PLUS) Conceptual Framework adapted from Glass and McAtee (2006); published in Brady et al. (2018).

The PLUS Consortium began its work by developing a research definition that would allow for the description of the current state of bladder health in U.S. adolescent and adult women across the lifecourse [11]. The consortium then developed a conceptual framework, which defined the broad levels of ecology that it would consider as spheres of influences on bladder health across the lifecourse. Levels of ecology included individual biology (e.g., bladder microbiome) and individual cognitive and behavioral factors (e.g., depression, fluid intake habits), interpersonal factors (e.g., peer norms), institutional factors (e.g., workplace and school policies regarding toilet access), and societal factors (e.g., laws governing access to healthcare; Figure 1) [12]. With this conceptual framework in place, the consortium began a process of identifying the most promising candidate risk and protective factors for bladder health in women. This process sought first to define the universe of risk and protective factors that one could investigate in the broadest possible sense and then to hone that macrocosm into specific research questions that the consortium would ultimately investigate.

In this manuscript, we describe the processes used to generate, iterate upon, test, and finally select factors for investigation, using the principles of design thinking as a framework to elucidate this process. Although the PLUS Consortium did not prospectively adopt design thinking as the organizing principle for this effort, this framework is well aligned with the process in which the PLUS Consortium engaged. We describe the lessons learned and how the consortium may have been further served by aligning more closely with design thinking principles, *a priori*. This case study may inform future prevention science research, particularly when a complex array of interrelated factors may contribute to health outcomes.

## 2. Methods

### 2.1. Overview of Design Thinking

The field of design began with the conceptualization and creation of material objects (e.g., appliances) and evolved to include the notion of constructing experiences (e.g., software user interface) [13]. Design thinking describes a set of principles and strategies by which innovations—broadly defined—are developed. The core tenets of design thinking include understanding end users’ desires, creating models to examine complex problems, developing prototypes to explore solutions, and tolerating failure as teams iterate on prototypes toward an optimal solution [14,15]. This approach has been used by corporate strategy teams, government services, and social change campaigns—and increasingly by health professionals—to solve complex problems [16,17,18,19]. Applying principles of design thinking to the development of research questions may likewise foster more comprehensive and innovative studies to inform prevention science and health promotion activities [13,14].

**Figure 2:**
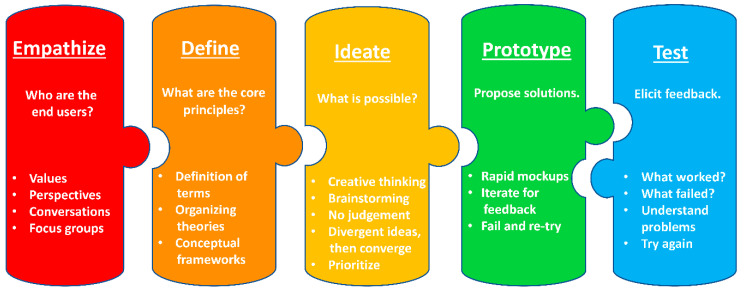
Stages of the design thinking process.

Design thinking is human-centered. It prioritizes building an understanding of the “end users” of the solution—i.e., the people for whom the solution is being created. When applied to the development of research questions, design thinking is aligned with strategies that engage different stakeholders, including scientific and lay communities [20,21]. Design thinking is also a creative process. Rather than being constrained by current practice and familiarity biases, design thinking asks innovators to visualize a world without accustomed limits to imagine entirely new solutions. Design thinking is aligned with the tenets of transdisciplinary science. It values working in multidisciplinary collaborative teams and gathering a wide range of perspectives. It iterates upon a comprehensive set of ideas to solve complex problems, using action-oriented rapid cycle prototyping of solutions (Figure 2) [13,14].

Design thinking is an iterative process consisting of five stages: (1) Empathize, (2) Define, (3) Ideate, (4) Prototype, and (5) Test [22]. The last two steps may be repeated many times until the optimal design has been achieved. During the *Empathize* stage, teams consider who the end user of the solution will be and what their core values and perspectives are to ensure the solution will be appropriate to their requirements. During the *Define* stage, teams consider the goals, skills available, and core principles that frame the work ahead. During the *Ideate* stage, teams value creative thinking and brainstorming as they generate the widest variety of possible solutions. With this foundational work accomplished, the *Prototype* and *Test* cycle begins, as teams undertake a process of creating possible solutions and then eliciting feedback on these potential solutions to shape and re-shape them until an optimal conclusion has been achieved [22].

The PLUS Consortium engaged in a thoughtful and iterative process to identify the risk and protective factors it would investigate as potential influences on bladder health for girls and women across the lifecourse. Below, we describe the development of the PLUS prevention research agenda within the framework of design thinking.

### 2.2. Empathize

In design thinking, the first stage of solving a problem is to empathize with the end-user of the solution. In our case, the immediate end-user of the solution (i.e., the research questions generated by this process) is the PLUS Consortium itself—and the ultimate end-users are the scientific and lay communities who are interested in, or affected by, the answers to the research questions generated. Thus, the NIDDK defined the immediate end users of these research questions when they chose investigator teams for the PLUS Consortium. Experienced investigators represented the interests of not only their disciplines, but also the many constituencies of patients and community members with whom they have worked and whose bladder health they have sought to understand and improve. Investigators had expertise across the lifecourse (e.g., adolescents, older adults), levels of ecology (e.g., interpersonal relationships, institutional structures), patient and community demographics (e.g., rural/urban regions, racial/ethnic groups, sexual/gender minorities), and risk and protective factors within different levels of ecology (e.g., microbiome, health behaviors, adverse childhood experiences, community characteristics, healthcare access).

Through weekly calls and quarterly in-person meetings, the consortium dedicated significant time to developing a common understanding of members’ core values and standpoints. Strategies to develop a common understanding included transdisciplinary engagement, educating membership about different research perspectives and content areas, and engaging in focused discussion that surfaced assumptions and viewpoints [10]. Members engaged in Myers–Briggs personality typing and facilitated sharing of types to enhance communication and empathy with respect to different work styles.

Further, the consortium dedicated time to understanding the broader community of adolescent and adult women’s core values and perspectives through community engagement activities and a large qualitative study (i.e., SHARE: Study of Habits, Attitudes, Realities, and Experiences of bladder health) [23]. Through this immersive process to empathize with the end-users of research questions, the consortium identified known and unknown needs and brought new insights to the work ahead [24]. This was essential to ensure the research questions developed would be appropriate to end users.

### 2.3. Define

The second stage of innovation in design thinking is to define the core principles that frame the work, synthesizing and incorporating what was learned in the Empathize stage. During the first year of its work together, consortium members developed a shared understanding of the principles of prevention science. Prevention science is based on the premise that effective health promotion necessitates an understanding of what leads to disease and how best to avoid precursors and exposures. To identify potential antecedents to human dysfunction and health (i.e., risk and protective factors), prevention scientists conduct etiologic studies that examine risk and protective factors within individuals (e.g., genes, cells, tissues, organs, multi-organ systems) and across the levels of social ecology surrounding individuals (e.g., family, peer group, school or workplace, healthcare system, neighborhood, community, society) [25,26,27,28]. Prevention scientists apply a life course developmental perspective to investigate how accumulated risk and protection lead to trajectories of health or disease [29,30,31,32]. Risk and protective factors identified through etiologic research become candidates for prevention efforts, which may include health promotion programs and changes to practices and policies within institutions and governments [33,34].

Candidate risk and protective factors considered by a research team are often constrained by the theories and practices that serve as resources for idea generation within disciplines represented on the team [35]. To further our goal of conducting *transdisciplinary* research, PLUS Consortium members developed a conceptual framework to organize potential risk and protective factors into areas of study that would facilitate the development of new, innovative research questions at multiple levels of biology and social ecology [12]. The PLUS Consortium conceptual framework was intentionally comprehensive, as it represented the entire ecology from which we could consider possible influences on women’s bladder health [12]. The consortium drew from separate, but complementary theoretical traditions and contemporary writings, including social ecological models, biopsychosocial models of health, Glass and McAtee’s Society–Behavior–Biology Nexus, and the World Health Organization’s conceptual framework for action on the social determinants of health (Figure 1) [27,28,34,36,37,38]. The consortium’s efforts toward defining the core principles of its work established the essential elements that would scaffold the solutions generated (i.e., the research questions).

### 2.4. Ideate

The third stage of the design thinking process is idea generation. During this stage, team members are encouraged to think broadly and move beyond the obvious to increase innovation. This is a time to challenge assumptions, provoke new ideas, and tolerate the unknown. During this stage, there is no attempt to evaluate ideas raised, establishing a safe space for creativity. The concepts generated through the ideation process both fuel the discovery process and provide the starting point for the generation of prototype solutions [22,39].

Once the PLUS Consortium had established a conceptual framework to bound its work, it engaged in a series of activities towards the generation and prioritization of research questions. The consortium convened in multiple rounds of subgroup configurations with defined conceptual tasks. The PLUS Terminology, Conceptual Frameworks and Models (TCFM) sub-committee—comprised of members representing each research center and the SDCC, as well as a diverse array of disciplines—began this ideation process. TCFM developed an initial list of risk and protective factors that could be studied within each of the following domains represented in the conceptual framework: (1) biology/body, (2) mind/behavior, (3) interpersonal relationships, (4) institutions, and (5) community and society [12].

To generate an even broader list of risk and protective factors of interest, the consortium implemented an ideation exercise with the entire consortium membership at an in-person meeting. Large sheets of paper were attached to the walls of the meeting space to represent five domains (i.e., levels of ecology). Consortium members were given colorful sticky notes and asked to think of as many candidate risk and protective factors for bladder health as they could. Consortium members circulated through the meeting space, discussed their factors with others, and affixed their sticky notes to the domain that best represented the factor, with some factors crossing domains. After this opportunity for the entire membership to contribute ideas, subcommittee members further developed the list to ensure that all risk and protective factors discussed had been captured. The TCFM subcommittee spent an additional month generating and collecting additional risk and protective factors of potential interest from consortium members, expanding the universe of potential factors under consideration for study.

By design, the interests of individual research members and research centers within the PLUS Consortium are broad. It is thus not surprising that the consortium generated over 600 potential risk and protective factors for potential study in this manner. While such an exercise and resulting product appeared overwhelming, this activity ensured that consortium members had considered all that was possible to study, including factors that may not have been considered within any single disciplinary group.

The Ideate stage of design thinking includes a space for both divergent (i.e., associative, creative) and convergent (i.e., focused, analytic) thinking [40]. Once a universe of ideas has been defined, these ideas must be filtered, narrowed, and synthesized into something that can be prototyped and tested. PLUS Consortium members next engaged in a convergent thinking activity to prioritize the potential 600+ risk and protective factors for study. This activity grouped consortium members by their research center and the SDCC (i.e., each funded site). Each site was asked to prioritize and rank order their “top 20” risk and protective factors for study. The criteria guiding this prioritization included (1) innovation/novelty, (2) potential for intervention/modifiability, (3) potential impact on public health, (4) contribution to health equity, (5) importance to stakeholders/at-risk populations, (6) feasibility for study, (7) ease of measurement, and (8) prevalence. This exercise was challenging and offered opportunities for sites to identify constellations of risk and protective factors of interest that could be grouped. For example, “toileting environment” encompassed a variety of specific factors of interest, including privacy, cleanliness, and safety. Similarly, “mental health” encompassed a variety of specific factors, including symptoms of attention deficit hyperactivity disorder, depression, and posttraumatic stress.

**Figure 3:**
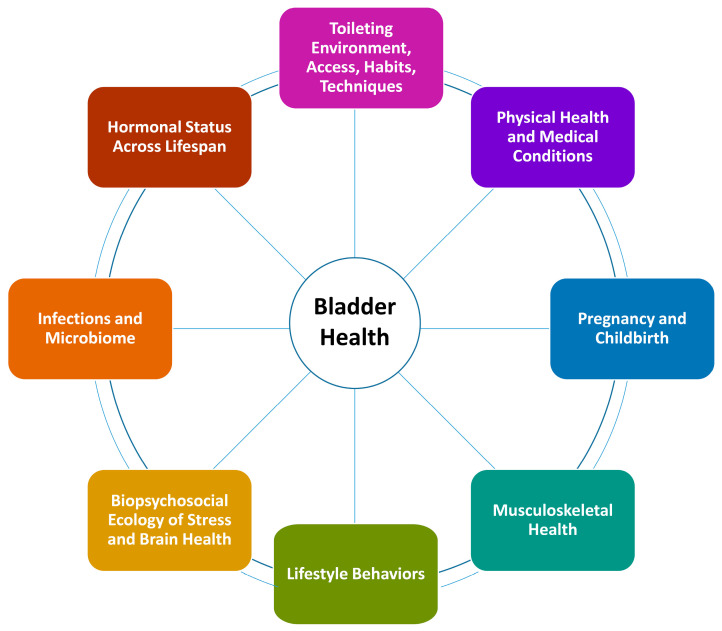
PLUS Consortium research themes.

Across the 7 research centers and SDCC, “top 20” lists were integrated to identify 44 unique constellations of over 400 factors ranked most important across the consortium (see Brady et al., 2018, Supplemental Appendix, for a complete list of prioritized factors). Prioritized risk and protective factors were classed further by the SDCC into 8 research themes: (1) biopsychosocial ecology of stress and brain health; (2) toileting environment, access, habits, and techniques; (3) pregnancy and childbirth; (4) physical health and medical conditions; (5) musculoskeletal health; (6) lifestyle behaviors; (7) infections and microbiome; and (8) hormonal status across the lifespan. A figure was created to depict themes as the spokes of a wheel, conveying that all themes were important to study (Figure 3). Within a research theme, risk and protective factors could be examined across all levels of biology and social ecology defined within the PLUS Consortium conceptual framework. Further, themes were considered across life stages to encourage the development of research questions incorporating a lifecourse perspective [29].

### 2.5. Prototype and Test

During the Prototype and Test stages, iterations of a solution are generated and tested quickly with a high tolerance for failure, prioritizing action and forward momentum [39]. The prototyping process surfaces problems and next solutions. It develops an end-product through risk-taking, failure, and pivoting until the desired result is achieved [13].

The consortium began prototyping its solution (i.e., a focused set of research questions) by dividing into two broad work groups to define how it would measure the 44 constellations of prioritized risk and protective factors in a national longitudinal observational study. The Individual Risk and Protective Factors work group focused on factors involving biology, the body, mind, and behavior, while the Environmental Risk and Protective Factors work group focused on factors involving interpersonal relationships, institutions, community, and society. These work groups were also divided methodologically, with the Individual Risk and Protective Factors work group focusing on measurement at the individual level (e.g., surveys, specimen collection, physical exams) and the Environmental Risk and Protective Factors work group focusing on direct measurement of the environment (e.g., review of employer toileting policies, observation of wait times or cleanliness of facilities, environmental scan of public restroom availability). During that time, consortium members divided the 44 constellations of prioritized risk and protective factors among small teams of investigators with relevant content expertise who worked to identify potential measures for each factor.

For a number of reasons, the consortium decided to pivot in a new direction seven months into this process. The consortium opted to restructure this work to examine risk and protective factors *across* all levels of ecology, rather than dividing *between* individual and environmental levels of ecology. Originally, the Environmental Risk and Protective Factors work group had been tasked with considering influences at the interpersonal, institutional, community, and societal levels. However, this became overwhelming as it was difficult for members of this group to remain focused on environmental-level measurement (e.g., direct observation), rather than individual-level measurement of environmental factors (e.g., perceived peer norms rather than actual norms, perceived control rather than actual environmental facilitators and constraints for toileting behaviors). Content expertise was divided across the two work groups for a given research theme (Figure 3), diluting effectiveness. Further, groups were considered too large to be nimble. Lastly, the consortium decided it was premature to consider measurement of risk and protective factors without having developed specific research questions that reflected these factors first, as the most appropriate measure for each factor might vary depending on the specific research question. For example, to examine the impact of physical activity on bladder health and LUTS, one could measure participation in high impact sports among adolescent women; prenatal and postpartum walking, running, and other forms of exercise among women across lifetime births; or a myriad of other forms of physical activity that are relevant during specific life stages and in specific contexts. Therefore, the consortium disbanded the Individual Risk and Protective Factors and Environmental Risk and Protective Factors work groups.

To develop and refine a more specific set of research questions, the consortium decided to assemble members into Theme Teams corresponding to the 8 research themes defined (Figure 3). Theme Teams were charged with developing specific research questions within their theme, considering all levels of ecology. To aid in the development of research questions, Theme Teams were encouraged, but not required, to develop conceptual models to depict the proposed mechanisms by which risk and protective factors could be associated with bladder health (e.g., mediation pathways) and the conditions under which such associations may occur (e.g., potential effect modification) [35]. It proved important to place risk and protective factors into the context of specific research questions to (1) ensure the biological or theoretical plausibility of proposed effects, (2) identify potential mechanisms or mediation pathways that could be explored in the national longitudinal observational study, and (3) identify overlapping mechanisms across research themes and questions to prioritize data collection. Having a better understanding of proposed mechanisms also aided in the selection of appropriate measures (e.g., type, duration, and timing of physical activity within a given life stage and context).

Theme Team activities resulted in the generation of 28 “working” research questions (i.e., prototypes) covering a broad range of candidate risk and protective factors for bladder health. Research question champions within Theme Teams presented their questions for the full consortium’s consideration (i.e., tested their prototypes) on two occasions. On the first occasion, they presented prototypes on a bi-weekly all-consortium call to allow members of other Theme Teams to weigh in on their developing research questions and to identify possible areas of synergy. On the second occasion, Theme Teams presented refined questions at an in-person meeting of the entire consortium.

To help the consortium evaluate the prototype questions, Theme Teams were given criteria for presentations. Theme Teams were to (1) identify the candidate risk and protective factors (i.e., independent variables) and outcomes of interest (i.e., dependent variable) within questions, (2) justify the importance of the question from the scientific literature, (3) assess feasibility for study in a longitudinal observational study of adolescent and adult women from the general population (i.e., prevalence of risk/protective factors, ability to assess by recall, requirement of any specific sample characteristics), and (4) determine whether their question required the development or validation of new measures or in-person measures (e.g., exam, biospecimen collection).

During the next in-person meeting of the entire consortium, Theme Teams presented refined questions. Theme Teams were asked to rank-order their presentation of questions by priority. The consortium’s Study Design and Methodology Group developed a list of possible scientific and practical considerations to assist Theme Team and consortium members in further prioritizing research questions (i.e., testing prototypes). Prioritization criteria facilitate impartial (as opposed to impassioned) and objective evaluation of questions and promote a transparent prioritization process guided by a common language and vision (e.g., considerations of greatest importance to the consortium). The list of considerations was refined by surveying consortium members about which considerations they considered to be of greatest importance for prioritizing research questions and by soliciting community input. Scientific and practical considerations clustered into 5 main themes: (1) significance (i.e., ultimate impact on bladder health), (2) proxy power (i.e., relatedness to other research questions), (3) feasibility of measurement, (4) public health relevance (i.e., possible impact beyond bladder health), and (5) uniqueness to PLUS (i.e., how well positioned the consortium versus other research groups would be to answer the question). In total, there were 23 considerations for each individual research question: 16 scientific (e.g., “If the research question relates to a modifiable factor, would the associated intervention be easy to implement/well-accepted? Low cost?”) and 7 logistic (e.g., “What is the length of time/number of items needed to address each construct?”), along with 2 additional considerations regarding the overall set of research questions (i.e., “Do our questions address all levels of our conceptual framework?” and “Do our questions address the full lifecourse?”). These considerations were presented to the full consortium at this meeting (Table 1).

Ahead of the next in-person meeting, the consortium conducted a consortium-wide survey asking investigators to assign each research question to 1 of 4 prioritization categories for a national longitudinal observational study, on the basis of scientific and practical considerations (Table 1). Categories included: (1) high priority to measure at baseline; (2) high priority, but not necessary at baseline; (3) low priority for the observational study; and (4) low priority for PLUS Consortium. At the in-person meeting, the results of the survey were presented and discussed. Most research questions garnered strong support by consortium members and were primarily distributed to categories 1 and 2. However, there was substantial variability; only 2 questions did not have anyone classify them as low priority. The consortium considered each refined research question (new prototypes) and whether corresponding measures should be included in the national longitudinal observational study, first through small group discussions and then in a consortium-wide discussion moderated by a trained professional moderator. Through this prototype testing process, the consortium selected research questions as relevant to the its future work and only one question was eliminated from consideration due to low support (i.e., “Do periodontal behaviors/status affect bladder health?”), leaving 27 prioritized research questions.

As the work of the consortium progressed and the design of the national longitudinal observational study began to take form, additional research questions began to be informally proposed (new prototypes), motivating the consortium to perform a final check to ensure that all important research questions had been identified. A survey was distributed to consortium members to request review of the 27 research questions and proposal of any additional, potentially important questions (testing). A total of 13 additional research questions were generated and prioritized in a similar manner as the original 27 questions. Two of these new questions were primarily distributed to categories 1 and 2, bringing our final tally of prioritized research questions to 29 (next prototype; Table 2). Although this step delayed progress to a small degree on the development of the observational study, it also provided consortium members with time to reflect on the full scope of their work and to identify important areas of investigation that had been omitted in the initial generation and narrowing of research questions.

The 29 research questions developed through this process were then taken up by the Study Design and Methodology Group for the planned national longitudinal observational study, ATTRIBUTES: Assessments Taken over Time: Relationships Influencing Bladder and Urinary Tract Experiences Study. The work of the Study Design and Methodology Group to continue refinement of questions and plan the national longitudinal observational study that will utilize these questions in some form is beyond the scope of this paper.

## 3. Discussion

### 3.1. Lessons Learned

We have described the process the PLUS Consortium used to identify the risk and protective factors it will investigate as potential influences on bladder health for women across the lifecourse. We mapped this process onto a design-thinking framework, which closely aligns with the strategies we employed. Although, we did not enter this process with an *a priori* intention to use a design thinking framework, we learned several lessons through this endeavor that may be instructive for other research groups aiming to identify a comprehensive and novel set of prevention science research questions in a new or neglected area of science.

Beginning with the intention of utilizing a design thinking process may have helped us to engage in this work more efficiently in several ways. While design thinking processes are iterative by nature, had we begun with this orientation, we would have re-ordered certain activities for better efficiency (Table 2). For example, it would have been ideal for the consortium to have engaged community stakeholders earlier in the process (at the Empathy stage) to inform the Ideation, Prototype, and Test stages of research question development. Although some consortium members were experienced in community engagement work, other members were less familiar with this field and reluctant to engage the community “too early” (i.e., before we had a clear path forward). Had we decided to use a design thinking perspective a priori, Empathy stage requirements would have forced the consortium to move more quickly to gather community perspectives and understandings in this earliest stage of design thinking. Our qualitative focus group study (i.e., SHARE) did not begin until July of 2017. Results required the consortium to revisit measure development work to ensure that community perspectives, language, and understanding of bladder health were incorporated appropriately. The consortium convened Rapid Assessment Partners (i.e., RAPs) of community members to gain feedback on particular issues. However, a broader, more systematic community engagement effort at the start of PLUS could have replaced some of these later efforts and better informed the Empathy stage.

We also discovered that it would have been helpful at the Define stage to have agreed upon a process for narrowing, selecting, and refining the risk and protective factors we would study. Without an agreed upon strategy, we cycled through several rounds and methods of narrowing down factors to converge on a focused set of research questions. We employed (1) individual consortium member ranking; (2) site level ranking; (3) group discussions, debates, and moderated deliberations; and (4) assessments of proposed questions using various criteria. These were useful exercises; however, had PLUS defined the considerations of greatest importance for the consortium in selecting risk and protective factors and formulating research questions, such criteria could have been used to promote a transparent and systematic prioritization process guided by a common language and vision. Without defined criteria in place to weigh the importance of different risk and protective factors and assess our prototype questions, we struggled to come to consensus. Establishing a consensus was important because proposed research questions could not all be answered in a single data collection timepoint.

Additionally, some members of the consortium believed that it was better to begin with the identification of variables and measurement strategies and proceed to developing research questions (i.e., inductive reasoning), while other members believed that research questions should be developed first and variables and measurement strategies should follow, on the basis of these questions (i.e., deductive reasoning). This reflected an epistemic divide. Those investigators who favored inductive reasoning were mainly concerned about incomplete operationalization (i.e., failing to measure potentially relevant variables). Those investigators who favored deductive reasoning believed that theoretical frameworks and other conceptual tools would aid the consortium in identifying the most important risk and protective factors to measure. Because the process for narrowing the universe of risk and protective factors was not pre-defined, the consortium ended up beginning in one direction (i.e., inductive reasoning within the Individual and Environmental Risk and Protective Factors work groups) and then changing approaches seven months later (i.e., deductive reasoning within Theme Teams). It would have been advantageous to clarify the process that would be used to generate and test prototypes earlier, during the Define stage. For example, one approach would have been for Theme Teams to work on the development of research questions and corresponding conceptual models using deductive reasoning, while another work group could identify covariates and other “core” measures using inductive reasoning.

Similarly, while we had a process for surfacing risk and protective factors, we did not define a plan at the start for how key covariates would be identified and incorporated. In an effort to ensure that certain covariates would be incorporated, Theme Teams began to form less novel research questions that centered around covariates to ensure their inclusion in data collection. Had a process for identifying key covariates been defined earlier, Theme Teams would not have been constrained by concerns that they needed to incorporate key covariates into research questions. Further, Theme Teams were encouraged—but not required—to build conceptual models to accompany their research questions; this was because there were so many questions under consideration and it seemed a burdensome task to build conceptual models for questions that may or may not be prioritized. Had a prioritization process been in place earlier, a smaller set of questions with accompanying conceptual models could have been built to guide measurement efforts.

Although better alignment with a design thinking framework may have helped us consider some issues earlier in our timeline, we accomplished activities consistent with design thinking. The fact that the consortium attempted one strategy and then changed mid-stream to another strategy is well aligned with the design thinking notion of being willing to fail. Design thinking requires risk taking. Innovation is necessarily messy. Although we would recommend that prevention scientists and transdisciplinary science teams consider such questions *a priori*, it is an anticipated part of the design thinking process that one cannot anticipate everything and therefore must be willing to move forward boldly and start again as needed. Further, in each stage of the consortium’s work, investigators had the opportunity to work with many sets of colleagues, which helped to prevent the regeneration of new silos within the consortium. This allowed us to build a strong, resilient, adaptable transdisciplinary team.

### 3.2. Limitations

Design thinking provided a useful framework to describe our process. However, there are limitations to its use. This approach may be challenging at first for health researchers and the scientific community, who typically take a linear approach to problem solving. Training in design thinking methodology is not readily available to health professionals and scientists. Further, design thinking is process that requires time and commitment to investigate and understand users’ needs, define what is critical, explore entirely new ideas, experiment with those ideas, and iterate. It requires time to fail and start again. It can be challenging to carve the space and freedom to engage in such a process. Nonetheless, this approach holds a great deal of promise to inform prevention science and transdisciplinary research when combined with rigorous scientific standards and methodologies.

## 4. Conclusions

Prevention science seeks to uncover multi-level risk and protective factors for health and disease that can be used to inform later interventions. Beginning with a human-centered approach that considers social and environmental contexts of end-users positions scientists to better utilize the knowledge they gain for health promotion practice. Using a design thinking process can be a useful strategy to generate a comprehensive and novel set of research questions. This may be particularly valuable in areas of prevention science where less work has been done to identify multi-level determinants of health.

Good research must begin with good research questions. Achieving consensus on which questions to prioritize when addressing complex health issues can be challenging. Applying principles of design thinking to the development of research questions may facilitate fruitful collaboration, yield transdisciplinary frameworks to understand complex phenomena, and clarify ideas of how to maximize co-learning to develop effective public health solutions.

## Figures and Tables

**Table 1:** Prioritization considerations for research questions.

**Scientific Considerations**	1. Does the research question involve a modifiable factor (i.e., does it inform prevention)? Primordial: Does it involve a risk/protective factor that can be prevented/promoted in unexposed individuals? Primary: Does it involve a risk/protective factor that can be modified to reduce/enhance its influence in exposed individuals? Secondary: Does it involve a risk/protective factor that can be used for screening women with early LUTS?
	2. If the research question relates to a modifiable factor, would the associated intervention be easy to implement/well-accepted? Low cost?
	3. Does the research question inform groups more or less susceptible to the influence of a particular risk or protective factor (i.e., effect modification)?
	4. Does the research question involve a risk or protective factor with a high prevalence (i.e., potential for high population attributable risk/impact)?
	5. Does the research question have the potential for policy level impact?
	6. Is the research question timely?
	7. Does the research question relate to a risk/protective factor specific to bladder health (not general health)?
	8. Does the research question help us understand women’s health, health behaviors, and health decision-making more broadly (i.e., does it have implications beyond bladder health?)?
	9. Is the research question supported by a plausible mechanism (e.g., biological, psychological, etc.)?
	10. Does the research question address a novel risk/protective factor?
	11. Does the research question provide confirmatory data for a less well-established factor?
	12. Does the research question provide confirmatory data for a more well-established factor?
	13. Is the research question important enough that any result (positive, inverse, null) will move the field forward?
	14. Could the research question push the field into new directions of inquiry or novel areas of research?
	15. Is the research question well-positioned for the uniqueness of the consortium, or is it better suited to an individual research group or to existing data/ongoing studies?
	16. Does the research exemplify transdisciplinary science?
**Practical Considerations**	17. Can the research question be addressed in a sample of the general population or does it require recruitment of a specific population?
	18. How many constructs are needed to answer the research question?
	19. What is the degree of invasiveness of assessment of each construct (e.g., self-report, physical examination, recall/real-time assessment)?
	20. What is the length of time/number of items needed to address each construct?
	21. Do any of the necessary constructs contribute to answering more than one research question?
	22. Does the research question overlap with multiple themes?
	23. Will the research question provide preliminary data for additional grants/pilot projects?
Regarding the overall pool of research questions: Do the questions address all levels of our conceptual framework? Do the questions address the full life course?	

**Table 2:** Lessons learned: Sequence of activities and proposed revisions for future research.

	**Dates**	**PLUS Consortium Activities**	**Proposed Revisions to Sequence or Timing**
**Empathize**	7/15-	PLUS investigators bring diverse clinical/community experience	
	7/15-	Remote calls and in-person meetings with transdisciplinary investigators	
	9/15-	Webinars share transdisciplinary knowledge and perspectives	
	9/15	Myers–Briggs type sharing	
	7/17–4/18	SHARE Qualitative Study	This activity would have been more helpful to begin earlier. It began during Prototyping and Testing, requiring revisiting of measures. A broad community engagement process early in this phase would have been preferable.
**Define**	7/15	U.S. National Institute of Diabetes and Digestive and Kidney Diseases defines inclusion in PLUS	
	9/15–7/17	PLUS develops research definition of bladder health	
	7/15–9/15	PLUS establishes conceptual framework	
**Ideate**	9/16–10/16	Terminology, Conceptual Frameworks and Models (TCFM) sub-committee generates list of candidate risk and protective factors	
	11/15	Consortium participates in sticky notes ideation exercise: 600 factors identified	This could have preceded the TCFM generation of factors.
	11/15–12/15	TCFM refines list of factors	
	1/16–2/16	Members rank top 20 determinants; 44 prioritized factors across 8 themes	
**Prototype and Test**	1/17–7/17	Individual and Environmental Work Groups develop measures for risk/protective factors	Consensus on a process to formulate research questions and measure of risk and protective factors at the Define stage would have prevented shifting directions.
	8/17–6/18	Theme Teams develop research questions	Consensus on a plan to determine key covariates would have prevented investigators from trying to incorporate key covariates into questions to ensure their inclusion, leading to more novel questions.
	10/17 and 11/17	Theme Teams present research questions to entire consortium for comment	
	11/17	Study Design and Methodology Group (1) defines criteria to prioritize research questions and (2) gathers consortium feedback about which scientific and practical considerations are most important for prioritization	It would have been helpful at the Define stage for the PLUS Consortium to have determined the scientific and practical considerations of greatest importance to the consortium. These criteria could have been used to prioritize and select risk and protective factors and research questions of highest importance efficiently through a transparent and objective process.
	4/18–2/19	PLUS reviews 27 questions and generates and prioritizes missing questions in a similar manner	
	2/19	PLUS adopts 29 research questions for refinement and use in a national longitudinal observational study	
